# Supplementary material for: Analysis of PPARγ Signaling Activity in Psoriasis
Source: Int J Mol Sci. 2021 Aug 10;22(16):8603. doi: 10.3390/ijms22168603 (PMC8395241; doi:10.3390/ijms22168603)
Supplement: Supplementary file 1 [file ijms-22-08603-s001.zip › Supplemental materials_Analysis of PPARg signaling activity in psoriasis/Pathway models/Models images and html files/Model of PPARG related pathways in psoriasis (short version)/6471.html]

IRAK1


# Protein IRAK1

|  |  |
| --- | --- |
| URN | urn:agi-llid:3654 |
| Total Entities | 0 |
| Connectivity | 2116 |
| Name | IRAK1 |
| Description | interleukin-1 receptor-associated kinase 1 |
| Notes | This gene encodes the interleukin-1 receptor-associated kinase 1, one of two putative serine/threonine kinases that become associated with the interleukin-1 receptor (IL1R) upon stimulation. This gene is partially responsible for IL1-induced upregulation of the transcription factor NF-kappa B. Alternatively spliced transcript variants encoding different isoforms have been found for this gene. [provided by RefSeq, Jul 2008] |
| Primary Cell Localization | Cytoplasm |
| Class | Protein kinase |
| ObjectType | Protein |

---

|  |  |
| --- | --- |
| Pathway | Osteoarthritis |
|  | Fatty Acids Block TLR Signaling in Enteroendocrine Cell |
|  | I-cell: CCK-8 Secretion and Regulation of Eating Behavior |
|  | Toll-like Receptors Act through MYD88-TIRAP Signaling |
|  | Antiviral Signaling through Pattern Recognition Receptors |
|  | Toll-like Receptors Act through MYD88 Signaling |
|  | TLR4 Signaling in Leukocytes |
|  | Th1-Cell Differentiation |
|  | Th17-Cell Differentiation |
|  | Toll-like Receptors in Sterile Inflammation |
|  | Toll-like Receptor Independent Sterile Inflammation |
|  | MC1R in anti-Inflammatory Signaling |
|  | Basophil Activation |
|  | Eosinophil Survival by Cytokine Signaling |
|  | Mast-Cell Activation without Degranulation |
|  | Mast-Cell Activation without Degranulation through IL33/IL1RL1 Signaling |
|  | Mast-Cell Activation without Degranulation through IL1R1 and TLR Signaling |
|  | Macrophage M1 Lineage |
|  | CD16/CD14 Proinflammatory Monocyte Activation |
|  | AHR Signaling in M1 Macrophages Function |
|  | anti-Inflammatory Function of Macrophage M2 Lineage |
|  | AXL Receptor Inhibits Macrophages and Dendritic Cells Function |
|  | Neutrophil Recruitment and Priming |
|  | Negative Acute Phase Proteins Synthesis |
|  | Positive Acute Phase Proteins Synthesis |
|  | Vascular Endothelial Cell Activation by Cytokines |
|  | Vascular Endothelial Cell Activation by Blood Coagulation Factors |
|  | Adipokines Production by Adipocyte |
|  | IL1B Expression Targets -> Nociception |
|  | Dendritic Cells Function in Atherosclerosis |
|  | RAGE/AGER and S100 Proteins in Cardiovascular Injury |
|  | Th1-Cells Activation and Proliferation in Atherosclerosis |
|  | Myocardial Infarction |
|  | Lymphocyte Mediated Myocardial Injury in Myocarditis |
|  | Toll-like Receptors (TLR) in Antiviral Innate Immune Response |
|  | Inflammatory Reaction in Acne Vulgaris |
|  | Keratinocyte Proliferation in Acne Vulgaris |
|  | Mast-Cells Activation in Atopic Dermatitis |
|  | Dendritic Cells Function in Psoriasis |
|  | Apoptotic Keratinocytes Clearance Recession in Systemic Lupus Erythematosus |
|  | Dendritic Cell Activation in Systemic Lupus Erythematosis |
|  | Th17-Cell Function in Systemic Lupus Erythematosus |
|  | Neutrophil and Macrophage Function in Systemic Lupus Erythematosus |
|  | Vitiligo |
|  | T-Cell Cytotoxic Response against Melanocytes in Vitiligo |
|  | Plasmacytoid Dendritic Cell in Diabetes Mellitus Type 1 |
|  | TLR2 Signaling in Treg-Cell in Type 1 Diabetes (Animal Model) |
|  | Toll-like Receptors in beta-Cell |
|  | TLR7 and TLR9 in Plasmacytoid Dendritic Cell |
|  | Conventional Dendritic Cell Role in Diabetes Mellitus Type 1 |
|  | Insulin Resistance in Myocytes Induced by Obesity |
|  | Hepatic Stellate Cells in non-Alcoholic Fatty Liver Disease |
|  | beta-Cell Destruction through Cytokines in Diabetes Mellitus |
|  | beta-Cell Destruction in Diabetes Mellitus Type 1 |
|  | Adipokines Production by Adipocyte Impaired in Obesity |
|  | CD4+T-Cell Response in Celiac Disease |
|  | Celiac Disease |
|  | Crohn's Disease |
|  | Dendritic Cell Dysfunction in Crohn's disease |
|  | Dendritic Cell Function in Ulcerative Colitis |
|  | Epithelial Cell in the Innate Immune Response in Ulcerative Colitis |
|  | Nicotine anti-Inflammatory Effect in Ulcerative Colitis |
|  | Antiphospholipid Antibodies in Endothelial Cell |
|  | B-Cell Chronic Lymphocytic Leukemia |
|  | Diffuse Large-B-Cell Lymphoma |
|  | Diffuse Large-B-Cell Lymphoma ABC Subtype |
|  | Proteins Involved in Hodgkin Lymphoma |
|  | Mucosa-Associated Lymphoid Tissue (MALT) Lymphoma |
|  | Splenic Marginal Zone Lymphoma |
|  | Antiphospholipid Antibodies and Endothelial Cell Interaction |
|  | IL33 Signaling Related Eosinophilia in Asthma |
|  | TSLP Signaling in Bronchial Epithelial Cell in Asthma |
|  | Club-Cell Role in Asthma |
|  | Basophil Activation in Asthma |
|  | Antigen-Presenting Cell Role in Asthma |
|  | Th17-Cell Differentiation in Asthma |
|  | Mucin Production in Goblet Airway Epithelial Cells |
|  | CFTR Facilitates Oxidative Stress in Airway Epithelium in CF |
|  | Airway Epithelial Cell Dysfunction |
|  | CFTR Expression in Epithelial Cells (Class I Mutations) |
|  | Osteoclast Activation in Gout |
|  | Neutrophil Recruitment in Sinovium in Gout |
|  | Monocytes Function in Gout |
|  | TNF and IL1B Induce Metalloproteinase Synthesis in Osteoarthritis |
|  | IL1B Induced Arthralgia |
|  | Dendritic Cell Dysfunction in Psoriatic Arthritis |
|  | Th17-Cell and Th1 Immune Responsein Psoriatic Arthritis |
|  | Osteoclast Activation in Psoriatic Arthritis |
|  | Synovial Fibroblast Activation in Psoriatic Arthritis |
|  | Osteoclast Activation in Rheumatoid Arthritis |
|  | Synovial Fibroblast Activation by Citokines in Rheumatoid Arthritis |
|  | TLR2 Induced Synovial Fibroblast Activation in Rheumatoid Arthritis |
|  | Autocrine Cytokine/Chemokine Loops in Systemic Scleroderma |
|  | Th17-Cell Numbers Reduction in HIV |
|  | Toll-like Receptors (TLR) in Periodontitis |
|  | IL1 Receptor Antagonist (IL1RN) Deficiency (DIRA) |
|  | PAPA Syndrome |
|  | TNF Receptor Associated Periodic Syndrome |
|  | Mevalonate Kinase Deficiency |
|  | Blau Syndrome |
|  | IL36 Receptor Antagonist (IL1F5) Deficiency (DITRA) |
|  | Cryopyrin (NLPR3) Associated Periodic Syndromes |
|  | Familial Cold Autoinflammatory Syndrome 2 |
|  | Familial Mediterranean Fever |
|  | Majeed Syndrome |
|  | Gluten Impact on Neuronal System (Hypothesis) |
|  | Microglia Activation in Alzheimer's Disease |
|  | HMGB1 and IL1B in Neuroinflammation |
|  | Microglia Activation in Parkinson's Disease |
|  | Subepithelial Fibroblasts in Middle Ear Cholesteatoma |
|  | Cholesteatoma |
|  | HPV E6 and E7 Induced Disruption of TNFR/NF-kB Signaling in Keratinocytes |
|  | Cytokines Trigger Otitis Media |
|  | MUC5AC Expression by NTHi and S. Pneumoniae |
|  | Immune System Role in Otitis Media |
|  | Alveolar Macrophage Negative Regulation Declined in COPD |
|  | Alveolar Macrophages Dysfunction in COPD |
|  | T-Cell Cytotoxic Activation |
|  | Alveolar Epithelial Cell Dysfunction in COPD |
|  | Mucin Hyperproduction in Goblet and Mucous Cells |
|  | Endothelial Cell Dysfunction in Glomerulonephritis |
|  | Toll-like Receptors in Pyelonephritis |
|  | Endothelial Cell Dysfunction in Pyelonephritis |
|  | NFKB Canonical Signaling Activation in Cancer |
|  | STAT3 and NFkB Activate Inflammation-Induced Tumorigenesis |
|  | NFKB Signaling Activation by Blocking of Tumor Suppressors |
|  | IDO1 in Cancer Immune Escape |
|  | IL1R -> STAT3 Signaling |
|  | IL1R -> NF-kB Signaling |
|  | NF-kB Canonical Signaling |
|  | TLR4 -> AP-1 Expression Targets |
|  | TLR5 Expression Targets |
|  | TLR7 Expression Targets |
|  | TLR4 -> AP-1/EGR1/HIF1A Expression Targets |
|  | TLR1 -> 2/6 Expression Targets |
|  | IL1B Expression Targets |
|  | TLR4 -> NF-kB/IRF Expression Targets |
|  | IL1A Expression Targets |
|  | TLR9 Expression Targets |
|  | TLR4 -> IRF Signaling |
|  | TLR4/5/7/9 -> NF-kB Signaling |
|  | TLR1/2/6 -> NF-kB Signaling |
|  | TLR -> AP-1 Signaling |
|  | pathway 4 |
|  | Model of PPARG signaling in psoriasis |
|  | Model of PPARG related pathways in psoriasis (short version) |
|  | New Pathway (3) |
|  | dsRNA induces innate immunity and INFs in alveolar cells |
|  | Innate immunity in Leukocytes |
|  | AGER feed forward loop in innate immunity |
|  | Dendritic Cell Activation in COVID19 |
|  | 1\_Inflammation in keratinocytes in the pathology of acne |
|  | 2\_Acute phase initiation in atopic dermatitis |
|  | 1\_TNF and IL1B provoke ECM degradation in osteoarthritis |
|  | 2\_Chondrocyte death in osteoarthritis |
|  | 1\_The role of Toll-like receptors in pyelonephritis |
|  | 2\_The role of endothelial cells in pyelonephritis |
|  | 1\_Insufficient activation of immune response in the middle ear epithelium cells in otitis media |
|  | 2\_Pathogens stimulate mucins expression in the middle ear |
|  | 3\_Arterial wall inflammation and calcification in atherosclerosis |
|  | 1\_Cigarette smoke causes dysfunction and production of inflammatory mediators in alveolar epithelial cells |
|  | 3\_Role of alveolar macrophages in pulmonary tissue destruction in COPD |
|  | 1\_Th2 cell response in asthma |
|  | 3\_1\_Mucus accumulation in asthma |
|  | 2\_2\_Eosinophilia and neutrophilia in asthma: eosinophil apoptosis |
|  | 4\_Mucin hyperproduction in goblet and mucous cells in COPD |
|  | 2\_Paneth cell dysfunction in Crohn's disease |
|  | 1\_Defects in response to pathogens in the gut promotes inflammation in Crohn's disease |
|  | 2\_Intestine epithelial cells drive immunological responses in ulcerative colitis |
|  | 1\_Intestine epithelial cell dysfunction in ulcerative colitis |
|  | 2\_Lipotoxicity in NAFLD |
|  | 1\_CD4+ T cell and epithelial cell response in celiac disease |
|  | 5\_Human papilloma virus E6 and E7 proteins promote epithelial cell proliferation |
|  | 4\_3\_Insulin resistance: FFA-related insulin resistance |
|  | 1\_CFTR expression and degradation in epithelial cells in cystic fibrosis (class I-II mutations) |
|  | 1\_Antigen-presenting cells promote islet dysfunction and immune system activation in T1D |
|  | 3\_Islet beta-cell destruction in T1D |
|  | 2\_Neuron and Schwann cell death |
|  | Figure 4\_Diabetes mellitus Type 1: conventional dendritic cell |

---

|  |  |
| --- | --- |
| MedScan ID | 3654 |

---

|  |  |
| --- | --- |
| LocusLink ID | 3654 |
|  | 16179 |
|  | 363520 |
|  | 102844 |

---

|  |  |
| --- | --- |
| Alias | interleukin-1 receptor-associated kinase-1b |
|  | interleukin IL1 receptor ssociated kinase I |
|  | IRAK |
|  | interleukin-1 receptor-associated kinase I |
|  | IL-1 receptor-associated kinase (IRAK) 1 |
|  | IL1rak |
|  | mPLK |
|  | Pelle (Drosophila) homolog |
|  | Irak1 |
|  | interleukin IL1rak |
|  | PELLE-like |
|  | OTTHUMP00000026020 |
|  | IL1 receptor associated kinase I |
|  | pelle |
|  | interleukin-1 receptor-associated kinase (IRAK) 1 |
|  | IL1R-associated kinase (IRAK) 1 |
|  | interleukin IL1 receptor ssociated kinase 1 |
|  | IL1R associated kinase 1 |
|  | Plpk |
|  | IRAK1b |
|  | RGD1563841 |
|  | pelle-like protein kinase |
|  | IL1R associated kinase I |
|  | Pelle homolog |
|  | IL1 receptor associated kinase 1 |
|  | OTTMUSP00000020306 |
|  | IRAK1-S |
|  | RP23-252M19.7 |
|  | OTTMUSP00000020303 |
|  | OTTMUSP00000019684 |
|  | OTTHUMP00000026014 |
|  | IRAK I |
|  | AA408924 |
|  | interleukin-1 receptor-associated kinase 1 |
|  | IRAK-1 |
|  | IL1 receptor ssociated kinase 1 |
|  | interleukin-1 receptor-associated kinase |
|  | interleukin-1 receptor-associated kinase-1 |
|  | IL1 receptor ssociated kinase I |
|  | Irak1\_predicted |
|  | Pelle homolog |

---

|  |  |
| --- | --- |
| EC Number | 2.7.11.1 |
|  | 2.7.1.- |

---

|  |  |
| --- | --- |
| GO ID | 0005524 |
|  | 0004704 |
|  | 0031072 |
|  | 0016301 |
|  | 0046982 |
|  | 0042803 |
|  | 0004672 |
|  | 0004674 |
|  | 0007254 |
|  | 0002755 |
|  | 0000187 |
|  | 0007250 |
|  | 0007568 |
|  | 0034605 |
|  | 0071456 |
|  | 0071222 |
|  | 0019221 |
|  | 0045087 |
|  | 0070498 |
|  | 0035556 |
|  | 0031663 |
|  | 0032088 |
|  | 0043066 |
|  | 0070423 |
|  | 0043123 |
|  | 0043406 |
|  | 0051092 |
|  | 1901224 |
|  | 1904996 |
|  | 0048661 |
|  | 0045893 |
|  | 0032481 |
|  | 0046777 |
|  | 0051259 |
|  | 0006468 |
|  | 0001959 |
|  | 0070555 |
|  | 0032496 |
|  | 0007165 |
|  | 0034134 |
|  | 0034142 |
|  | 0034162 |
|  | 0002224 |
|  | 0007178 |
|  | 0060337 |
|  | 0016032 |
|  | 0005737 |
|  | 0005829 |
|  | 0010008 |
|  | 0045323 |
|  | 0005811 |
|  | 0005634 |
|  | 0005886 |
|  | 0005149 |
|  | 0000166 |
|  | 0016740 |
|  | 0002376 |
|  | 0090370 |
|  | 0045892 |
|  | 0016310 |
|  | 0043507 |
|  | 0032494 |
|  | 0016020 |
|  | 0005622 |
|  | 0034146 |
|  | 0006916 |
|  | 0035872 |
|  | 0051403 |
|  | 0002756 |
|  | 0005515 |
|  | 0016567 |
|  | 0034138 |
|  | 0035666 |
|  | 0008063 |
|  | 0034130 |
|  | 0048011 |
|  | 0034166 |
|  | 0038123 |
|  | 0045944 |
|  | 0016772 |
|  | 0038124 |
|  | 0004842 |
|  | 0016563 |
|  | 0032582 |
|  | 0005624 |
|  | 0004872 |
|  | 0008152 |
|  | 0005625 |
|  | 0045941 |
|  | 0000287 |
|  | 0007249 |
|  | 0010874 |
|  | 0002237 |
|  | 0019901 |

---

|  |  |
| --- | --- |
| KEGG ID | hsa:3654 |
|  | mmu:16179 |
|  | rno:363520 |

---

|  |  |
| --- | --- |
| MedLine Reference | 10191251 |
|  | 10383454 |
|  | 10395652 |
|  | 10395695 |
|  | 10723722 |
|  | 10854325 |
|  | 10899313 |
|  | 11076863 |
|  | 11096118 |
|  | 11287640 |
|  | 11397809 |
|  | 11518704 |
|  | 11701612 |
|  | 11744690 |
|  | 11777917 |
|  | 11828002 |
|  | 11937546 |
|  | 11953430 |
|  | 11960013 |
|  | 11976320 |
|  | 12034707 |
|  | 12055225 |
|  | 12138165 |
|  | 12242293 |
|  | 12370331 |
|  | 12391239 |
|  | 12477932 |
|  | 12496252 |
|  | 12566447 |
|  | 12619925 |
|  | 12721283 |
|  | 12724322 |
|  | 12856330 |
|  | 12860405 |
|  | 12874243 |
|  | 12925853 |
|  | 14625308 |
|  | 14684752 |
|  | 14752294 |
|  | 15069085 |
|  | 15084582 |
|  | 15465816 |
|  | 15489334 |
|  | 15489336 |
|  | 15523691 |
|  | 15647277 |
|  | 15691841 |
|  | 15767370 |
|  | 15829295 |
|  | 16024789 |
|  | 16052631 |
|  | 16094384 |
|  | 16107720 |
|  | 16203735 |
|  | 16286016 |
|  | 16381901 |
|  | 16477006 |
|  | 16690127 |
|  | 16831874 |
|  | 17053167 |
|  | 17276401 |
|  | 17382928 |
|  | 8599092 |
|  | 8837778 |
|  | 9286695 |
|  | 9371760 |
|  | 9374458 |
|  | 9625770 |
|  | 9918122 |
|  | 10190904 |
|  | 10224059 |
|  | 10349636 |
|  | 10673339 |
|  | 10754329 |
|  | 10922068 |
|  | 11042159 |
|  | 11076861 |
|  | 11217851 |
|  | 12054681 |
|  | 12418963 |
|  | 12466851 |
|  | 12626533 |
|  | 12819136 |
|  | 12855817 |
|  | 12860565 |
|  | 14660668 |
|  | 14673102 |
|  | 15102471 |
|  | 15292196 |
|  | 15555532 |
|  | 15782199 |
|  | 15849359 |
|  | 15896328 |
|  | 16046709 |
|  | 16141072 |
|  | 16141073 |
|  | 16247769 |
|  | 16917541 |
|  | 17477969 |
|  | 8663605 |
|  | 8889548 |
|  | 8905588 |
|  | 9530638 |
|  | 9625767 |
|  | 15793310 |
|  | 15890643 |
|  | 15946255 |

---

|  |  |
| --- | --- |
| Organism | Homo sapiens {Organism urn:agi-taxid:9606} |
|  | Mus musculus {Organism urn:agi-taxid:10090} |
|  | Rattus norvegicus {Organism urn:agi-taxid:10116} |
|  | Homo sapiens |
|  | Mus musculus |
|  | Rattus norvegicus |

---

|  |  |
| --- | --- |
| Mouse chromosome position | X 37.61 cM |
|  | X 29.6 cM |

---

|  |  |
| --- | --- |
| OMIM ID | 300283 |

---

|  |  |
| --- | --- |
| Rat chromosome position | Xq37 |
|  | 1 |
|  | X |

---

|  |  |
| --- | --- |
| Hugo ID | 6112 |
|  | HGNC:6112 |

---

|  |  |
| --- | --- |
| Human chromosome position | Xq28 |

---

|  |  |
| --- | --- |
| Swiss-Prot Accession | P51617 |
|  | D3YTB5 |
|  | P51617.2 |
|  | B1AUW6 |
|  | Q8BR10 |
|  | Q540G0 |
|  | Q62406 |
|  | Q62406.3 |
|  | B2RYH5 |
|  | D3DWW3 |
|  | D3DWW4 |
|  | Q7Z5V4 |
|  | Q96C06 |
|  | Q96RL2 |
|  | B1AUW4 |
|  | Q6Y3Z5 |
|  | Q6Y3Z6 |
|  | Q8K4N5 |
|  | Q8K4N6 |
|  | Q8K4N7 |
|  | Q8K4N8 |
|  | Q99J34 |

---

|  |  |
| --- | --- |
| PIR ID | G02512 |

---

|  |  |
| --- | --- |
| GenBank ID | NC\_000023 |
|  | XM\_005274668 |
|  | XP\_005274725 |
|  | NM\_001025243 |
|  | NP\_001020414 |
|  | NM\_001025242 |
|  | NP\_001020413 |
|  | NM\_001569 |
|  | NP\_001560 |
|  | NG\_008387 |
|  | AF030876 |
|  | AAC08756 |
|  | CH471172 |
|  | EAW72760 |
|  | EAW72761 |
|  | EAW72762 |
|  | EAW72763 |
|  | EAW72764 |
|  | EAW72765 |
|  | HF583647 |
|  | CCQ43144 |
|  | KT584727 |
|  | U52112 |
|  | AF346607 |
|  | AAK62888 |
|  | BC014963 |
|  | AAH14963 |
|  | BC054000 |
|  | AAH54000 |
|  | BE276817 |
|  | DQ054788 |
|  | AAY88246 |
|  | L76191 |
|  | AAC41949 |
|  | P51617 |
|  | NC\_000086 |
|  | NM\_001177976 |
|  | NP\_001171447 |
|  | NM\_001177974 |
|  | NP\_001171445 |
|  | NM\_001177973 |
|  | NP\_001171444 |
|  | NM\_001177975 |
|  | NP\_001171446 |
|  | NM\_008363 |
|  | NP\_032389 |
|  | AB088390 |
|  | BAC06196 |
|  | BAC06197 |
|  | BAC06198 |
|  | AC091472 |
|  | AF121351 |
|  | AL672002 |
|  | CH466650 |
|  | EDL29846 |
|  | EDL29847 |
|  | EDL29848 |
|  | EDL29849 |
|  | EDL29850 |
|  | AB088370 |
|  | BAC05683 |
|  | AF103876 |
|  | AAD13224 |
|  | AK009132 |
|  | AK045958 |
|  | BAC32547 |
|  | AK052933 |
|  | AK143694 |
|  | BAE25502 |
|  | AK144879 |
|  | AK183779 |
|  | AK196997 |
|  | AK198649 |
|  | AK203259 |
|  | AK210148 |
|  | AY184362 |
|  | AAO63012 |
|  | AY184363 |
|  | AAO63013 |
|  | AY184364 |
|  | AAO63014 |
|  | BC004778 |
|  | AAH04778 |
|  | BP760087 |
|  | BY729815 |
|  | U56773 |
|  | AAC52694 |
|  | Q62406 |
|  | NC\_005120 |
|  | NM\_001127555 |
|  | NP\_001121027 |
|  | XM\_006229598 |
|  | XP\_006229660 |
|  | AC\_000069 |
|  | AAHX01006903 |
|  | AC106169 |
|  | CH474099 |
|  | EDL84996 |
|  | BC166780 |
|  | AAI66780 |
|  | ACE86439 |
|  | EU831520 |
|  | NW\_003871103 |
|  | CAL38089 |
|  | EU831427 |
|  | ABBA01045321 |
|  | ABBA01045322 |
|  | AC\_000042 |
|  | ACE87114 |
|  | CAM18731 |
|  | CAM18730 |
|  | AAHY01212722 |
|  | AC\_000155 |
|  | CAM18729 |
|  | NC\_018934 |
|  | XP\_005277899 |
|  | CAM18740 |
|  | CAM18737 |
|  | CAM18736 |
|  | CAM18739 |
|  | CAM18738 |
|  | CAM18733 |
|  | CAM18732 |
|  | XM\_011531158 |
|  | CAM18735 |
|  | CAM18734 |
|  | AM392624 |
|  | NC\_005100 |
|  | XM\_005277842 |
|  | CAL37502 |
|  | AMYH02040852 |
|  | AABR06006958 |
|  | XP\_011529460 |
|  | AM393211 |
|  | NT\_167198 |
|  | NW\_001842419 |
|  | NW\_927732 |
|  | NG\_007107 |
|  | AC\_000066 |
|  | CR604638 |
|  | CR611861 |
|  | CR619624 |
|  | Q96C06 |
|  | NT\_039706 |
|  | NW\_001035173 |
|  | Q540G0 |
|  | Q8BR10 |
|  | Q8K4N5 |
|  | Q8K4N6 |
|  | Q8K4N7 |
|  | Q8K4N8 |
|  | Q99J34 |
|  | NW\_001084770 |
|  | NW\_048052 |
|  | NT\_011726 |
|  | XM\_001057078 |
|  | XP\_001057078 |
|  | XP\_343845 |
|  | NT\_039745 |
|  | XM\_343844 |
|  | 117645246 |
|  | 117646774 |
|  | 119593166 |
|  | 119593167 |
|  | 119593168 |
|  | 119593169 |
|  | 119593170 |
|  | 119593171 |
|  | 121945341 |
|  | 121947255 |
|  | 1220313 |
|  | 14485012 |
|  | 15929005 |
|  | 3002590 |
|  | 32450332 |
|  | 68235820 |
|  | 74751786 |
|  | 8928535 |
|  | Q0JTS3 |
|  | Q0JVF8 |
|  | 123122658 |
|  | 123122659 |
|  | 123122660 |
|  | 123122661 |
|  | 123122662 |
|  | 12409196 |
|  | 13435858 |
|  | 148697899 |
|  | 148697900 |
|  | 148697901 |
|  | 148697902 |
|  | 148697903 |
|  | 15214058 |
|  | 21907906 |
|  | 21952766 |
|  | 26337723 |
|  | 29119242 |
|  | 29119244 |
|  | 29119246 |
|  | 4235482 |
|  | 74150742 |
|  | 81866794 |
|  | 81866795 |
|  | 81866796 |
|  | 81866797 |
|  | 81872982 |
|  | 81874791 |
|  | 81903008 |
|  | BE949425 |
|  | BG074046 |
|  | BG076768 |
|  | AA408924 |

---

|  |  |
| --- | --- |
| Swiss-Prot ID | IRAK1\_HUMAN |
|  | IRAK1\_MOUSE |
|  | D3DWW3\_HUMAN |
|  | B2RYH5\_RAT |
|  | Q96C06\_HUMAN |
|  | Q540G0\_MOUSE |
|  | B1AUW6\_MOUSE |
|  | Q8BR10\_MOUSE |
|  | D3DWW4\_HUMAN |

---

|  |  |
| --- | --- |
| Cell Localization | Cytoplasm |
|  | Nucleus |
|  | Lipid droplet |

---

|  |  |
| --- | --- |
| Ensembl ID | ENSG00000184216 |
|  | ENSP00000377291.2 |
|  | ENST00000393687.6 |
|  | ENSP00000358991.2 |
|  | ENST00000369974.6 |
|  | ENSP00000358997.3 |
|  | ENST00000369980.8 |
|  | ENSP00000392662.2 |
|  | ENST00000429936.6 |
|  | ENSMUSG00000031392 |
|  | ENSMUSP00000033769.8 |
|  | ENSMUST00000033769.14 |
|  | ENSMUSP00000064448.5 |
|  | ENSMUST00000068286.11 |
|  | ENSMUSP00000109994.3 |
|  | ENSMUST00000114354.9 |
|  | ENSMUSP00000110000.3 |
|  | ENSMUST00000114360.9 |
|  | ENSMUSP00000109992.1 |
|  | ENSMUST00000114352.7 |
|  | ENSRNOG00000060869 |
|  | ENSRNOP00000070832.1 |
|  | ENSRNOT00000092207.1 |
|  | ENSMUSP00000033769 |
|  | ENSP00000377287 |
|  | ENSMUST00000114360 |
|  | ENSRNOT00000065177 |
|  | ENST00000393687 |
|  | ENST00000393682 |
|  | ENSRNOP00000061751 |
|  | ENSRNOT00000092207 |
|  | ENSP00000358991 |
|  | ENSRNOP00000070832 |
|  | ENSP00000392662 |
|  | ENST00000369980.7 |
|  | ENSP00000358997 |
|  | ENSMUST00000033769 |
|  | ENSMUSP00000064448 |
|  | ENST00000429936 |
|  | ENST00000369980 |
|  | ENSMUST00000068286 |
|  | ENSMUSP00000109994 |
|  | ENSMUSP00000109992 |
|  | ENSMUSP00000110000 |
|  | ENST00000369974 |
|  | ENSRNOG00000037263 |
|  | ENSMUST00000114354 |
|  | ENSMUST00000114352 |
|  | ENSP00000377291 |

---

|  |  |
| --- | --- |
| MGI ID | MGI:107420 |
|  | 107420 |
|  | 2147821 |

---

|  |  |
| --- | --- |
| RGD ID | 1563841 |

---

|  |  |
| --- | --- |
| Unigene ID | Hs.522819 |
|  | Rn.22238 |
|  | Mm.38241 |

---

|  |  |
| --- | --- |
| Homologene ID | 37496 |

---

|  |  |
| --- | --- |
| Shape | Sickle-vertex |

---

|  |  |
| --- | --- |
| IPI ID | IPI00293652 |
|  | IPI00474424 |
|  | IPI00472717 |
|  | IPI00385328 |
|  | IPI00125866 |
|  | IPI00940703 |
|  | IPI00060149 |
|  | IPI00950547 |
|  | IPI00114859 |
|  | IPI00752328 |
|  | IPI00640278 |
|  | IPI00647563 |
|  | IPI00646527 |
|  | IPI00646038 |
|  | IPI00879385 |
|  | IPI00894401 |
|  | IPI00652700 |
|  | IPI00828277 |
|  | IPI00828308 |
|  | IPI00828350 |
|  | IPI00890138 |
|  | IPI00828379 |
|  | IPI00830416 |
|  | IPI00828949 |
|  | IPI00828905 |
|  | IPI00830776 |
|  | IPI00896173 |
|  | IPI00776724 |

---

|  |  |
| --- | --- |
| Homo sapiens Chromosome position | Xq28 |

---

|  |  |
| --- | --- |
| Ensembl Transcript ID | ENST00000369980 |
|  | ENST00000369974 |
|  | ENST00000393687 |

---

|  |  |
| --- | --- |
| FunctionalClass | ATP binding |
|  | NF-kappaB-inducing kinase |
|  | magnesium ion binding |
|  | nucleotide binding |
|  | protein binding |
|  | protein homodimerization |
|  | protein kinase binding |
|  | protein serine/threonine kinase |
|  | receptor |
|  | transcription activator |
|  | transferase |
|  | interleukin-1 receptor binding |
|  | kinase |
|  | protein kinase |

---

|  |  |
| --- | --- |
| Mus musculus Chromosome position | X 29.6 cM |
|  | X |

---

|  |  |
| --- | --- |
| KEGG pathway | Apoptosis |
|  | Toll-like receptor signaling pathway |

---

|  |  |
| --- | --- |
| Rattus norvegicus Chromosome position | Xq37 |

---

|  |  |
| --- | --- |
| Ensembl Gene ID | ENSG00000184216 |

---
